# Application and Extension of the Alcohol Recovery Narratives Conceptual Framework

**DOI:** 10.1177/10497323231197384

**Published:** 2023-09-08

**Authors:** Mohsan Subhani, Usman Talat, Holly Knight, Joanne R. Morling, Katy A. Jones, Guruprasad P. Aithal, Stephen D. Ryder, Stefan Rennick-Egglestone

**Affiliations:** 1 Nottingham Digestive Diseases Biomedical Research Centre (NDDC), School of Medicine, 6123 University of Nottingham, Nottingham, UK; 2 NIHR Nottingham Biomedical Research Centre, 6123 Nottingham University Hospitals NHS Trust and the University of Nottingham, Nottingham, UK; 3 Alliance Manchester Business School, 5292 University of Manchester, Manchester, UK; 4 Population and Lifespan Sciences, 6123 University of Nottingham, Nottingham, UK; 5 School of Medicine, Applied Psychology, 6123 University of Nottingham, Nottingham, UK; 6 School of Health Sciences, Institute of Mental Health, 6123 University of Nottingham, Nottingham, UK

**Keywords:** alcohol misuse, narrative inquiry, conceptual framework, recovery narrative, recovery story, validation, narrative interviews

## Abstract

Recovery narratives are personal stories of health problems and recovery. A systematic review proposed a conceptual framework characterising alcohol misuse recovery narratives, consisting of eight principal dimensions, each with types and subtypes. The current study aims to apply and extend this preliminary conceptual framework. Semi-structured interviews were conducted to collect alcohol misuse recovery narratives from adult participants. A two-stage inductive and deductive thematic analysis approach was used to assess the relevance of the dimensions and types included in the preliminary conceptual framework and identify new components. The sample consisted of 11 participants from diverse socioeconomic backgrounds who had previously displayed varying degrees of alcohol misuse. All conceptual framework dimensions (genre, identity, recovery setting, drinking trajectories, drinking behaviours and traits, stages, spirituality and religion, and recovery experience) were present in the collected narratives. Three dimensions were extended by adding types and subtypes. Whilst the existing conceptual framework fitted the collected narratives, a new dimension describing the *alcohol environment* was required to fully characterise narratives. Types included in the *alcohol environment* dimension were *policy and practice* and *social dynamics*. The extended framework could guide the production of resources enabling clinicians to engage with narratives shared by their clients.

## Introduction

In mental health research, recovery narratives have been defined as personal stories presenting internal or external events that occur over time, which describe adversity or struggle and self-defined strength, success, or survival (Llewellyn-Beardsley et al., 2019). They can be presented in a range of forms, including video, audio, prose, and poetry (McGranahan et al., 2019). They are frequently shared with others (Rennick-Egglestone et al., 2019b). The sharing of recovery stories is a phenomenon present across a range of treatment programs for alcohol misuse, such as Alcoholics Anonymous (AA) (Lederman & Menegatos, 2011). For alcohol misuse, recovery can be defined as a period of sustained abstinence from alcohol, improvement in social and employment functioning, and mental and physical health (Witkiewitz et al., 2019). Personal stories have been used by humans to share adversities such as alcohol misuse which can help to reappraise and achieve recovery (Weegmann & Piwowoz-Hjort, 2009). Sharing narratives can motivate personal recovery and open opportunities for working on positive behaviour change by encouraging and supporting oneself and others (Witkiewitz et al., 2019).

Narrative approaches are well-known in healthcare research (Barton, 2008; Haas, 2012; Jordens et al., 2001). A broad range of lived experience narratives have been studied, of which recovery narratives are just a subset. Researchers collect and analyse narratives describing experiences of health and illness, allowing for the intimate, in-depth study of an individual’s experiences over time and in context (Clandinin & Caine, 2013). Recovery narratives have been studied across a range of health concerns and can provide insights into the phenomenology of recovery (Zwerenz et al., 2017).

Repeated studies across a broad range of populations have demonstrated the formation of a recovery narrative can promote mental and physical health in the narrator (Pennebaker & Seagal, 1999). A growing evidence base suggests recovery narratives can be used by healthcare practitioners as an intervention to support patients’ recovery, for example, through the inclusion of recovery-oriented autobiographies in psychotherapeutic practice (Boylstein et al., 2007; Sommer, 2003). Recovery narratives have been used in stroke rehabilitation, where they can help patients to reconstruct their identity and to re-orientate themselves towards the possibility of recovery post-stroke (Boylstein et al., 2007). Representing a similar function, sharing a recovery narrative in an AA session can help an individual reorient their sense of identity towards maintaining sobriety (Lederman & Menegatos, 2011). Sharing narratives can help patients make informed choices on selecting a specific treatment and can improve compliance (Yaskowich & Stam, 2003). Moreover, sharing personal accounts of illness publicly can lead to increased social awareness and reduced stigma both for the narrator and the recipient, can contribute to changes in health policy, and can improve understanding of research (Sharf, 2001).

Recovery-orientated interventions are becoming more popular in drug and alcohol treatment settings (Neale et al., 2015). However, there remains an ongoing debate in contemporary literature concerning the optimal definition of recovery (Best et al., 2016). Some researchers focus on recovery as a relatively objective phenomenon that can be empirically measured across various domains of functioning. Others see recovery as a purely subjective phenomenon that is heterogeneous and reveals underlying idiosyncrasies of the recovery process (Best et al., 2016). Our work is aligned with this latter definition; we have turned to recovery narratives to understand and document these idiosyncrasies and hence to develop a conceptual understanding of their characteristics. In addition, being valuable to alcohol misuse recovery research, a clear understanding of the characteristics of alcohol misuse recovery narratives could optimise their use in clinical practice and maximise their positive impact (Llewellyn-Beardsley et al., 2020).

A conceptual framework describing the characteristics of alcohol misuse recovery narratives has previously been developed through a systematic review and narrative synthesis (Subhani et al., 2022). The review identified a rich source of existing literature describing the characteristics of alcohol recovery narratives. The review synthesised characteristics into eight overarching dimensions, each containing specific types and subtypes. Dimensions identified in the Alcohol Recovery Narratives Conceptual Framework (ARNCF) were genre, identity, recovery setting, drinking trajectories, drinking behaviours and traits, stages, spirituality and religion, and recovery experience. The ARNCF synthesises characteristics identified in analyses of narratives collected from 1055 participants across 32 empirical studies.

The ARNCF was produced with the intention of facilitating the research community to interpret and develop knowledge from alcohol recovery narratives, by introducing concepts, which can be adopted, modified, or challenged. This is in keeping with a tradition of treating conceptual frameworks as legitimate knowledge products in research, for example, as advocated by Blumer (1954) (in relation to conceptualisations of social settings) and Jabareen (2009). A better conceptual understanding of the characteristics of alcohol recovery narratives as a genre can enable the identification of knowledge gaps (e.g. around particular types of narrative) and inform the development of new behavioural interventions making use of narratives. It can also provide a framework to facilitate structured conversations between clinicians and patients to improve behavioural compliance and reduce stigma (Manning, 2006).

The current study aims to apply the ARNCF to a new corpus of alcohol misuse recovery narratives generated through semi-structured interviews. The objectives are (a) to evaluate the fit of the ARNCF to this corpus, (b) to identify candidate extensions, and (c) to explore individual experiences within the sample.

## Methods

The study was conducted as part of ‘The Knowledge of Liver Fibrosis Affects Drinking (KLIFAD) feasibility randomised control trial’. Ethical approval was obtained from the West of Scotland Research Ethics Service on 20 January 2021, REC reference: 20/WS/0179. The RCT was prospectively registered (ISRCTN16922410), and the trial protocol was published (Subhani et al., 2021). All procedures were in accordance with the ethical standards of the responsible committee on human experimentation (institutional and national) and with the Helsinki Declaration of 1975, as revised in 2000. Informed consent was obtained from all patients for being included in the study.

The KLIFAD trial is investigating the feasibility of integrating transient elastography (TE) into community alcohol services to facilitate early diagnosis of liver disease and promote positive change in addictive behaviours (Subhani et al., 2023). Fibrosis is the medical term for the scarring of the liver. TE is a type of ultrasound technology that measures the degree of stiffness in the liver caused by this scarring. Alcohol misuse recovery was defined as ‘a deeply personal, unique process of change, a way of living a satisfying, hopeful and contributing life even with limitations caused by illness [and] a process involving the development of new meaning or purpose in one’s life’. This drew on prior conceptualisations of recovery and recovery narratives developed by mental health researchers (Anthony, 1993; Rennick-Egglestone et al., 2019a).

### Participants

Inclusion criteria were as follows: (a) aged 18 years and over, (b) has been diagnosed with alcohol use disorder (AUD) or alcohol-related liver disease (ARLD), (c) self-identifies as ‘recovered’ or ‘being in recovery’ from their condition (as per recovery definition provided above and in supplemental material (S1 Text)), (c) has previously received one or more transient elastographies, (d) able to give informed consent, and (e) willing to be video recorded.

Exclusion criteria were as follows: (a) known to have any other liver disease apart from ARLD and (b) has a primary substance misuse other than alcohol.

Participants were purposively identified for variation in age, gender, ethnicity, and liver stiffness (Coyne, 1997), prioritising variation in liver stiffness as assessed at initial TE. They were recruited through existing patient forums at community alcohol services, by offering information to patients self-presenting to any of the trial treatment settings, and by snowball methods. A maximum variation sample on liver stiffness was sought, covering self-reported TE scores indicating normal, intermediate, and advanced liver stiffness (Benoot et al., 2016). Details of the recruitment process have been reported (Rennick-Egglestone S, 2023).

### Procedure

The following variables were collected on case report forms at the start of face-to-face interviews: age, gender, ethnicity, peak alcohol intake, living arrangement, occupation, current drinking status, length of sobriety, and liver stiffness measure (LSM).

#### Qualitative Semi-Structured Interviews

Qualitative semi-structured interviews were conducted by the lead researcher (MS). Each participant took part in a 30- to 60-minute interview conducted in a clinical setting or at the participant’s usual place of living, as selected by the participant. A topic guide was drafted in consultation with a patient and public involvement (PPI) group consisting of members with personal experience of alcohol misuse and recovery. Following accepted practice in narrative research, the initial part of the topic guide comprised open-ended questions to elicit alcohol recovery narratives, with minimal or no interruption from the researcher so that the narratives produced were presented within the frame of reference of the participant (Llewellyn-Beardsley et al., 2020; Spector-Mersel & Knaifel, 2018). Through this initial question, participants were asked to narrate their alcohol recovery story over time with a beginning, middle, current situation, and future plans (Llewellyn-Beardsley et al., 2020; McAdams, 1993). A sample interview guide is in Table S1. All interviews were video recorded, transcribed, and pseudonymised.

Eleven participants were recruited. Two participants had a history of homelessness; five were unemployed, four were working, and two were retired. One participant had engaged with AA. There were no narratives from people identifying as LGBTQIA2S+.

### Analysis

Transcribed interviews were uploaded to NVivo version 12. To maintain anonymity, the name of participants, third parties, institutions, and places were either redacted or replaced with a unique identifier. A two-stage qualitative thematic approach was used (Spector-Mersel & Knaifel, 2018). The initial coding frame was derived from the ARNCF (Subhani et al., 2022). Two researchers (MS and UT) independently coded and analysed the data at the sentence and paragraph levels.

The deductive and inductive analysis consisted of the following steps: (a) organisation of data, (b) obtaining the general sense of narratives, (c) coding of narratives, (d) identifications of themes and subthemes, and (e) data interpretation and compilation of results (Butina, 2015). Through stages d and e, the authors also attended to the contents of coded transcript fragments and used this material to develop narrative descriptions of phenomena relevant to alcohol misuse recovery processes.

In the first stage, narratives were analysed deductively, using dimensions and types from the preliminary Alcohol Recovery Narratives Conceptual Framework (ARNCF) as codes (Table 1) (Subhani et al., 2022). This was to assess the fit of the existing ARNCF to the data.

**Table 1.** Alcohol Recovery Narratives Conceptual Framework (ARNCF).

Dimensions	Types			
Genre	Drama	Redemption	Drinking tale	Identity tale
Identity	Renewal	Construction	Formation	
Recovery setting (positioning)	Recovery within treatment	Recovery outside treatment		
Drinking trajectory	Upward	Fluctuating	Steady	Downward
Drinking behaviours and traits	Non-alcoholic	Alcoholic	Personality traits	
Stages (sequence)	Origin of difficulty	Episode of change	Recovery	Ongoing struggle
Spirituality and religion	Religion versus spirituality	Belonging		
Recovery experience	Positive	Negative		

The dimensions and types from the original ARNCF were used as part of the coding process.

In the second stage, the interview material not covered by the existing conceptual framework was inductively categorised into new codes. These codes were later classified as either new dimensions or types, thereby extending the ARNCF. The approach from the original systematic review was adapted to develop a preliminary frame organising new themes. Two authors (MS and UT) identified new themes and produced a preliminary framing presenting these themes. The preliminary framework was reviewed, and relationships between entities in the framework were explored. The agreed new themes were added to the pre-existing ARNCF.

Coder 1 (MS) completed the initial coding, created a duplicate file, and removed code names. Coder 2 (UT) coded this file independently. Coders then discussed the codes to reach an agreement, and any conflicts were resolved through discussion with the senior author (SRE). The process was supervised by two researchers (SRE and HK), including monitoring the transparency of coding and addressing any potential conflict.

Where possible, the language used by participants was preserved while maintaining the clarity of analysis. The overarching CHIME (connectedness, hope, identity, meaning, and empowerment) framework (Best & Lubman, 2012; Slade et al., 2014) was also used.

### Examination of Other Characteristics

Sub-group analyses were conducted to explore the impact of specific characteristics, the subgroups were predetermined a priori from the previously published systematic review (Subhani et al., 2022). These were (a) gender, (b) history of homelessness, and (c) presence of mental health problems. The two-stage analysis process was repeated to assess the validity of ARNCF for these different characteristics.

## Results

Mean age was 56.0 years (SD 9.9 years), six (54.5%) participants identified as male and five (45.5%) as female. Ten identified as white and one with a minority ethnicity. Median peak alcohol intake was 190 units per week (range 105–300). The length of sobriety ranged from 3 years to over 10 years. The median LSM was 14.3 (range 3.6–27) kilopascal (kPa).

### Comprehensiveness of Alcohol Recovery Narratives Conceptual Framework (ARNCF)

Eight dimensions from the ARNCF were present in all narratives (Table 2). The type *‘spirituality versus religion’* was not identified in any narratives, and six participants expanded on *‘belonging’*.

**Table 2.** Comprehensiveness of Alcohol Recovery Narratives Conceptual Framework (ARNCF).

Participant	Dimensions							
ID	Genre	Identity	Recovery setting	Drinking trajectory	Drinking behaviours and traits	Stages (sequence)	Spirituality and religion	Recovery experience
P1	Yes	Yes	Yes	Yes	Yes	Yes		Yes
P2	Yes	Yes	Yes	Yes	Yes	Yes	Yes	Yes
P3	Yes	Yes	Yes	Yes	Yes	Yes	Yes	Yes
P4	Yes	Yes	Yes	Yes	Yes	Yes	Yes	Yes
P5	Yes	Yes	Yes	Yes	Yes	Yes		Yes
P6	Yes	Yes	Yes	Yes	Yes	Yes	Yes	Yes
P7	Yes	Yes	Yes	Yes	Yes	Yes		Yes
P8	Yes	Yes	Yes	Yes	Yes	Yes		Yes
P9	Yes	Yes	Yes	Yes	Yes	Yes	Yes	Yes
P10	Yes	Yes	Yes	Yes	Yes	Yes	Yes	Yes
P11	Yes		Yes	Yes	Yes	Yes		Yes

#### Dimension 1: Genre

All four types (drama, redemption, drinking tale, and identity tale) were present. Nine participants had redemptive narratives. In the *‘drinking tale’* type, the subtypes painful past, reinforcement, loss of uniqueness, relationship with oneself, and helping others were present in all narratives. In the *‘identity tale’* type, the subtypes ‘stages of life’, ‘sex’, and marginalised societies were present, whereas sexual orientation was not identified. Illustrative examples from participants’ interviews are given in Table 3.

**Table 3.** Illustrative Examples for Genre.

	Quotes from narratives
**Drama**	
Comedy theatre	I still learn stuff everyday about alcohol, how to hide it, how to disguise your drinking, stuff like that. And it’s pretty funny some of it, like ‘oh I wish I’d thought of that’ you know?
Quest	That’s when we disappeared for three-four years, we went our own way and I said to ‘A’ I said ‘right, it’s me, you and “B,” we’re going to sort this out in our head’ and then further down the line we decided to try and help people through things like this.
Melodrama	‘The checking, I get anxious, I get thoughts, I’ve got negative thoughts going on in my head. I think I-I, you know, I get that silly side all the time, but I just get really worked up and wound up by things’. ‘I think what a lot of it is, is people keep telling how talented, I get told that I taught “C” everything he knows’.
**Redemption**	
Redemptive	‘I’ve got to be careful right through now, erm, I was perhaps fairly fortunate’. ‘It was hard, it was hard. Erm, I didn’t want to die of liver disease, I don’t know if you’ve ever seen anyone, it’s not nice. I’ve got x children, I couldn’t. I thought I cannot, if that’s going to happen to me, I cannot have any of my family round me, I don’t want them to see me like that, yellow, bloated, horrible, I thought ‘I can’t do that’. And erm, I don’t want to die like that, it’s not nice, it’s slow, lingering, there’s nothing nice about it’.
Non-redemptive	Well, I think sometimes there bloody is, the problem has been the problem, the other people’s problem that think I’ve got a problem, you know what I mean.
**Drinking tale**	
	Find someone you trust and talk to them about it, don’t let them talk to you, you talk to them about it, because the last thing an alcoholic needs is somebody talking at them, they need to just take a step back, get them in a scenario where they will talk and let them talk because I think you’ll find, that within minutes they’ll come out with reasons why they actually drink a lot, and once they’ve said it, they’ve more or less been truthful to themselves, whereas if they weren’t given the opportunity to talk to somebody, they’ll carry on drinking.
Painful past	‘My husband divorced, and I got myself into a very bad relationship which was based on drink and that is when my drinking got serious’. ‘My dad died, and I just went through a really big bad patch when I was on holiday and started drinking more and more and it just had a big effect on me, and it’s just changed my life’. ‘I think erm, my friend who committed suicide but rang me up before he did it, that was the killer for me in terms of drinking, that was just like, I couldn’t cope, I really couldn’t cope with that’.
Reinforcement	I was perhaps fairly fortunate in that erm, it was a, it wasn’t a close friend but I played bowls with him, and he was diagnosed just maybe six months before me but he didn’t stop drinking and he’s not with us anymore and he used to give advice as well, he’d say ‘whatever they say to you H, just do it’, so I’ve tried to do that so support from that friend as well.
Loss of uniqueness	I’m just a regular person that likes Coro and Eastenders and things like that, erm, but also that regular person that’s lost a lot.
Improved relation with oneself	I’m quite an intelligent person as well and erm, I just it just got hold of me.
Helping other	Yeah, it does, honestly, it’s been the hardest time of my life and I’ve got another chance now, and I’m going to make the most of it, and if I can help anybody, anybody, I will, I will, I feel for all of them out there, I really do, it’s really cruel.

The quotes are taken from participants’ interviews.

#### Dimension 2: Identity

During their recovery journeys, participants went through different stages of identity transformation including ‘identity renewal’, ‘identity construction’, and ‘identity formation’. Participants shared emotions of guilt, shame, and hitting rock bottom. They went through cognitive restructuring to construct a new identity.

I just got to that real rock bottom that somehow, I just managed to stop. (P2)

Completely different, yeah, I have acceptance of myself today. I can’t believe, I can’t believe how the difference in myself, of my thinking, of who I am. In fact, even before I started drinking even you know err as a child up to my drinking, I am a different person, yeah. (P2)

Those friends that I met, voluntarily we set up an extra group and just people, I still see those people once a week every week now, three years on. (P11)

Eventually, the participants adapted to their new roles and reconstructed their social relationships. The multiple stages of identity transformation involve a shift in perspective that may be sustained through positively reinforcing cues such as social processing of emotions, change in recovery capital, and self-nurturing. This shift in perspective eventually leads to a change from addictive to non-addictive behaviour.

#### Dimension 3: Recovery Setting

Most of the participants apart from one (P11) had ‘recovery within treatment settings’. The most common institutions participants formally interacted with were community alcohol services, community alcohol detoxification units, primary care substance misuse clinics, Alcoholics Anonymous, mental health services, and secondary care hospitals. The participants described their interaction within treatment settings as positive and supportive towards recovery.

I went into that place in ….., and it was good, it was a structured. (P6)

I decided to, through the help of my GP and my liver specialist, I sought out a local group and I volunteered myself. (P8)

The participant who achieved ‘recovery outside treatment setting’ (P11) did not like group therapy, due to other group members telling them to stop drinking and change their behaviour. They disagreed with diagnosis labelling and believed that though they were drinking more than recommended, they were still in control.

I would read about it, I mean I always read what I could about being an alcoholic and how it creeps up on you and everything and about recovery and the twelve steps and sitting with a group of people saying, ‘I’m an alcoholic’ and I thought it’s not for me, I don’t want that. I actually ended up not saying anything cos I thought well I’m going to have to stop some of these people in order to say, ‘I have a fairly normal life, I just drink too much bloody alcohol and I want help stopping it’, but this isn’t helping me I’m afraid, so that was the end of that. I never had that breakthrough; I could certainly never sit in a group and say, ‘I’m an alcoholic’. (P10)

One person shared their experience of natural recovery, which represented an internal locus of control. The tendency to give control away to agents in the recovery setting may require the building of either trust over time with others or a self-driven attempt to come full circle with one’s addiction. This involves processing shame in some cases.

#### Dimension 4: Drinking Trajectory

In the drinking trajectory dimension, participants exhibited the *‘upward’*, *‘downward’*, and *‘fluctuating’ *trajectories, whilst the ‘steady’ drinking trajectory type was absent. Nine participants demonstrated a ‘slow’ upward trajectory, whereas two had a ‘sharp’ upward trajectory*.* Participants alluded to factors, which supplemented different types of trajectories. Specifically, in the context of how ageing relates to drinking habits and the ensuing impact on addiction, participants displayed a tendency to account for their narrated experiences in terms of changes in their social and personal circumstances. This suggests that recovery narratives involve the socioeconomic and highly personalised experience of ageing as a catalyst in piecing together the old identity one revisits, now, in recovery retrospectively. Participants talked about the impact of retirement, change in social responsibilities, and surplus income supplementing increases in alcohol intake, whereas a decline in physical health, enhanced family support, and a change in social capital supported a reduction in alcohol consumption.

I’d got my normal money going in, the mortgage being paid, normal things being looked after and then this bonus money, so of course, the amount that I was drinking went up quite steadily. (P7)

I’d started to drink a little more heavily I was what now is called an assistant head in a secondary school. (P5)

#### Dimension 5: Drinking Behaviours and Traits

At the time of interview, seven participants were abstinent, three had significantly reduced alcohol intake, and one participant was drinking in a controlled manner (as defined by UK Department of Health recommendations). In relation to the ARNCF, the following ‘personality traits’ were present: passive, prosocial, anti-social, and dishonest. The grandiose trait was not observed.

In the context of reviewed narratives, ‘prosocial’ and ‘passive’ personality traits supported addictive behaviours. Some participants began drinking due to peer pressure or in the form of companionship drinking, largely to maintain social acceptance. On the other hand, passive traits made participants vulnerable to abuse, exploitation, and manipulative practices by others, particularly where spousal relationships were involved. At the same time, dependence on alcohol promoted dishonesty and anti-social tendencies such as stealing, conflict, lying, and self-harm.

I used to be the, go to the pub on the way home from work, five o clock, couple of pints, you know with the mates and that kind of thing. (P1)

I took me to a part in a very dangerous, dark time where I was considering taking my own life, laid on tram tracks, stole from shops, stole bottles of wine. (P9)

#### Dimension 6: Stages (Sequence)

Most participants described recovery as a non-linear process. Common triggers for alcohol misuse were companionship drinking, occupational stressors, psychological trauma such as childhood abuse or toxic relationships, loss, grief due to the death of a family member or friend, mental health issues, and having a surplus income or surplus time. Participants described feelings of rejection, denial, acceptance, acknowledgement of the problem, and surrender eventually leading to a successful recovery. Examples of turning points were seeking help, having a near-death experience, being informed of having liver disease, changing from negative to positive social connections, self-realisation of excessive alcohol consumption, and observing others developing alcohol-related physical disease. Illustrative examples from participant interviews are given in Table 4.

**Table 4.** Illustrative Examples for Stages.

	Quotes from narratives
**Origin of difficulty**	
Start of drinking	I got divorced and then it just went, it spiralled downhill from then on.
	I think the killer for me was one of my friends rang me up, told me that he loved me and then committed suicide. And the first time I drank in the morning was at his funeral, that was the start.
Negative effect	I thought it could kill the pain but [sighs] the pain I was suffering, but no.
Drinking progress	We did have a lot of fun to be fair, but it just got a bit more and more and more.
Problems	I lost my license; I lost my job.
Drinking worsen	I would get a box of, I don’t know, ten, the fridge packs, and at first that would last a couple of nights, then it would last a night, so you’d need to get a bigger box and a bigger box.
**Episode of change**	
Blame and escape	But we were very honest with the doctor about how much we were drinking, and he never said ‘you need to stop that’.
Identification of problem	It was just my friend, she picked us up cos you know your just collapsing and you’re not able to do anything and you’re getting all worked up and I thought ‘ohh I’m fine’ and she said “no you’re not” is “looking after yourself”.
Alcoholic regression	It just gradually stopped, gradually.
Rejection and denial	I think the thing that I could never be about alcohol and as I say right up this minute is I’ve never been honest about it, I always denied that I had a problem because in my head I didn’t believe I had a problem.
Turning points	I went to see my GP and they thought I needed help and that’s when I got counselling and got support to just realise, I’ve got an issue.
**Recovery**	
Acknowledging problem	It meant that I’d got severe damage to my liver and that I had to be careful.
Surrender	I’ll be honest, when I gave up, obviously ‘A’ gave up and her liver failed, and she had that pain, but I made the decision to give up and I’ll be honest and truthful.
Acceptance	I think I drank all my emotions; I never had any acceptance of myself and erm, that’s different today, I’ve worked, I’ve just worked through all that.
Help	I think it’s admitting that you needed help and to grab that help was a big thing for me, especially because I’d, I’d had such a long sobriety before.
Become sober	I had that done in the March and then I actually stopped drinking in the April. So, and I’ve been three years sober now.
**Ongoing struggle**	
Being sober	I’m just, a small path, small path, I’m hoping that somehow it gets better. But it wasn’t as easy as that.
Maintaining sobriety	I’ve told all my friends, relatives, everyone that I’ve given up drinking, so I have no social pressure and I avoid any social occasions where I’m on my own, so I’m not tempted individually to have a drink.
Maintaining recovery	My friend had come up to visit me and I just had to be strong and just, I just decided, once you got through being detoxed, I just carried on, however hard that was, which it was, it was very hard to do, but err I managed to pull myself back up with help of other people of course.

The quotes are taken from participants’ interviews.

#### Dimension 7: Recovery Experience

Participants had a combination of *‘positive’* and *‘negative’* recovery experiences. The *‘positive’* type was predominant across the narratives. The commonly recurring positive recovery experiences were empowerment, improved relationship and family trust, finding lost opportunities, enjoying new activities, and improved mental health. Participants revised their roles to better reintegrate into local communities and felt happy to be alive. Some described ongoing experiences of wandering thoughts about their painful past, lost friends, and financial burden. Associated physical and mental health comorbidities such as fibromyalgia, anxiety, and depression made life harder.

#### Dimension 8: Spirituality and Religion

*‘Spirituality versus religion’* as a type was not identified in the narratives. Participants did talk about a sense of belonging. ‘*Belonging*’ as a type represented the adaptive feelings participants exhibited across key events to do with reintegration into society. In the *‘Belonging’* type, participants further shared experiencing ‘lack of belonging’, ‘a search for belonging’, and ‘attaining belonging’.

### Variation in Individual Experiences Within the Sample

#### Gender

Of all included participants, six identified as male and five as female. Those who identified as female were younger with a mean age of 51.2 years (SD +/−3.9) compared to males who had a mean age of 59.9 years (SD +/−10.6). Female participants had consumed a higher quantity of alcohol (units per week) at peak compared to males (mean 236.0, SD +/−55.9 vs 164.1, SD +/− 36.6). Female participants had higher liver stiffness measures (mean 15.2 kPa, SD +/− 8.6 vs 10.7 kPa, SD +/− 5.3). Some female narrators acknowledged the impact of harmful relationships with men as a trigger for alcohol misuse. Some female narrators described drinking behind closed doors to avoid social stigma and shame.

Like most of my problems in my life have been bad choices in men. I don’t really have much to do with men anymore. (Female)

I was never, I was a drinker behind closed doors, I was never a pub drinker or going out. I always used to do it in secret. (Female)

I thought all the things I knew that I shouldn’t be thinking I just thought I could just control it and, and I couldn’t. I think a lot of shame around that as well. (Female)

#### Homelessness and Alcohol Recovery

Two participants had personal experiences of homelessness. They had a shared history of a painful past. Both lacked a sense of belonging, making them vulnerable to addictive and anti-social behaviours.

I’ve been taken to hospital that many times, I’ve been arrested that many times, erm, I’d been in fights, I’d had black eyes, I was, I’ve been sectioned. It took me to a part in a very dangerous, dark time where I was considering taking my own life, erm, tried numerous occasions, laid on tram tracks, stole from shops, stole bottles of wine, and obviously when I was homeless. (P3)

For both, recovery was only made possible within a treatment setting by a multiagency integrated healthcare approach involving mental health, community alcohol, and social care services. As part of their recovery, these participants went through a significant identity shift from a non-functional person to a more functional and integrated member of society. Therefore, they found the path to recovery was hard but achievable within a holistic multidisciplinary framework.

I had that many fallbacks when I went through this process, it’s not easy, I’m not going to say it’s easy, nobody who has ever been in my position will ever say it’s easy, cos it’s not, it’s not. (P4)

#### Alcohol and Mental Health

Alcohol misuse and mental health problems were common themes across all the narratives. The analysis confirmed participants’ experienced low personal self-esteem, cognitive dissonance, and lack of love from others and a sense of belonging, denial, rejection, and shame. Poor mental health could be the cause or consequence of alcohol addiction.

I was very anxious, very anxious. I think on the outside I always looked confident, so I always had that anxiety and fear and I always used to. I think what it did looking back, it gave me confidence that I never had to be, you know, to be in those, I think socially I was quite awkward as well, so it gave me that ability to be more confident, yeah. (P2)

Participants frequently perceived alcohol as a medication to numb physical or psychological pain, suppress anxiety, and look more confident by suppressing internal conflict and insecurity.

I just feel what happened to me could really happen to anybody. It’s that alcohol is the best painkiller, is the best reliever of stress and anxiety that’s out there. (P3)

Common mental health problems reported by participants were anxiety, depression, post-traumatic stress disorder (often associated with childhood trauma or abusive relationships), obsessive–compulsive disorder (OCD), anti-social behaviours, lack of self-confidence, emotional instability, feeling on edge, and suicidal thoughts. Affective disorders were the most prevalent, and these disorders influenced participants’ construal of inter-personal reality driving and acted as a driver for alcohol addiction. Although participants narrated that input from mental health services was useful both in obtaining and maintaining recovery, they were put off by long waiting times and felt let down by the system.

They promised from a personal point of view, they said, ‘you get clean and stay sober for say 6 months, we’ll give you’ and I have this in writing actually, he gave me therapy/counselling that I need, and he said, ‘and you will have earned it’. Erm so I’m sober nearly three years and I’m still waiting, yeah, still waiting, so yeah, the system, I feel let down a bit by the system. (P11)

### Refinement of Alcohol Recovery Narratives Conceptual Framework (ARNCF)

Refinements were made to three dimensions: recovery settings, drinking trajectory, and drinking behaviour and traits. Based on themes identified across the interviews, a new dimension of ‘alcohol environment’ was added (Table 5).

**Table 5.** Refinement of Alcohol Recovery Narratives Conceptual Framework.

Dimensions	Types	
Recovery setting (positioning)	Recovery within treatment	
Pre-existing subtypes	AA narratives	
	Dual diagnosis narratives	
	Poly-drug abuse narratives	
New subtypes	Post hospitalisation narratives	
	Post non-invasive tests (NITs) for liver disease narratives	
Drinking trajectory	Triggers for upward trajectory	Turning points
	Retirement	Decline in physical health
	Kids living independently	Supporting partner
	Surplus income	
	Surplus time	
Drinking behaviours and traits	Personality traits	
Pre-existing subtypes	Anti-social	
	Prosocial	
	Passive	
	Dishonest	
Missing subtype	Grandiose	
New subtype	Avoidance behaviour	
Alcohol environment	Policy and practice	Social dynamics
	Affordability	Acceptability
	Availability	Impact of pandemic
	Exposure and advertisement	
	Constrained service	
	Opportunistic testing	
	Impact of pandemic	

The ‘recovery settings’ dimension originally represented the mode of recovery either within or out of formal treatment settings (former termed ‘natural recovery’). In the type ‘recovery within treatment settings’, there were initially three subtypes. Two further subtypes were identified: ‘post hospitalisation narratives’ and ‘post-non-invasive tests (NITs) for liver disease narratives. Hospitalisation and interaction with medical healthcare providers facilitated recovery.

You know I’m lying in that hospital bed in the liver unit absolutely scared stiff and I didn’t know why apart from reading the thing ‘liver unit’. So, during that night, it was praying and also realism and truth, I was there in bed that night and I was facing truth. My consultant, I said to him I said ‘what is it? What else can I do to get my liver better?’ he says, ‘you’re okay, well you’re not okay but’ he says, ‘because you’ve taken the problem away from your liver, you should be okay. (P3)

Moreover, additional non-invasive tests for liver disease conducted in primary or secondary care supported change in high-risk drinking behaviour. Participants indicated that knowledge of liver disease obtained through NITs supplemented a change in their drinking behaviour. In certain cases, participants preferred to have follow-up tests to see the progress they had made. Positive changes (e.g. lower scores at subsequent tests) induced feelings of reward, and lower stiffness scores were reassuring.

So started at 27, 29, 27, the next time I went back it was 24, that was six months later. Six months later 19, so there’s my motivation again ‘ohh it’s working, not drinking, my liver’s getting better, it’s getting softer. (P8)

I think it would have knocked me more the other way if it had been bad, definitely, because I knew what I was doing, even though I couldn’t stop. I think it, yes it would of, it would have had a different impact on me. (P1)

The ‘drinking trajectory’ dimension describes the impact of ageing on drinking but lacks the detail of the associated changes in socioeconomic circumstances. We observed that during the process of ageing, the following factors acted as a trigger to increase alcohol consumption: retirement, children transitioning to live independently, and having surplus income and time. However, a decline in physical health and a supportive partner acted as a turning point.

But eventually erm after I retired, I got depressed, I had nothing to do, it was horrible, and of course, you’re thinking about drinking. (P5)

I’d got my normal money going in, mortgage being paid, normal things being looked after and then this bonus money, so of course, the amount that I was drinking went up quite steadily. (P7)

The ‘drinking behaviour and traits’ as a dimension represented antecedents or consequences of alcohol misuse. We identified a new subtype which we have labelled ‘avoidance behaviour’ related to alcohol misuse. Participants possessing this trait found it difficult to manage others and found alcohol as a way to escape their responsibilities.

I knew it right from being a child that I couldn’t control other people, and I didn’t want to control other people, so internally I had a big conflict that each time I moved on I felt like I was moving away from the bit that I liked. (P7)

The current study provides information on ‘homelessness and alcohol misuse’, which hitherto remains absent in the previously proposed framework. For these participants, their recovery was only possible by an integrated multiagency approach due to the complexity of associated social and mental health issues.

#### New Dimension

*‘Alcohol environment’* as a dimension comprises the following types: ‘policy and practice’ and ‘social dynamics’. The policy and practice iteratively inform each other wherein the former relates to the generation of ideas followed by the latter, which typically covers implementation concerns. Here, it describes the impact of alcohol-related policies on addictive behaviours and subsequent recovery. Social dynamics as a type indicates the impact of change in social practice over time. Following subtypes were found: affordability, acceptability, availability, exposure and advertisement, constrained services, pandemic, and role of opportunistic testing for early detection of liver disease in populations at risk.

‘Affordability’ describes the buying capacity of a person to buy alcohol. One can consume more alcohol if purchasing power is increased or otherwise can turn to cheap alternatives if purchasing power decreases. ‘Acceptability’ describes the social acceptance of different drinking behaviours; specifically, our participants found that certain factors within a culture supported detrimental levels of alcohol consumption to the extent that was experienced as a normal behaviour validated by others. ‘Availability’ describes the ease of access to alcohol. Our participants narrated that over the years, alcohol has become easier to access due to policies such as changes in supermarket alcohol licensing and seasonal deals, allowing alcohol sales outside pubs with extended hours of business, and the adjacent propensity of drinking at home. ‘Exposure and advertisement’ describe how alcohol has been promoted through multimedia, at times ambiguously labelled or often lacking details concerning the harmful impact of alcohol on health. ‘Constrained services’ describes the pressure on health services to deal with alcohol misuse and associated mental health problems in a timely manner, which can result in reduced trust among service users. The ‘pandemic’ describes the impact of the coronavirus outbreak and imposed social restrictions on drinking behaviours. Participants described their alcohol consumption as increasing during the pandemic. ‘Early detection of liver disease’ describes the impact of having non-invasive tests (NITs) of liver disease and knowing the results. Participants narrated that knowing the results of transient elastography supported them to change addictive behaviour. The illustrative examples for ‘alcohol environment’ as a dimension are given in Table S2.

## Discussion

All conceptual dimensions were present in collected narratives, with three of these dimensions extended by adding types and subtypes. A new dimension ‘alcohol environment’ was added to the framework, and this functions to discern and identify broader policy level as well as nested individual level factors driving various patterns of alcohol misuse. During their recovery, participants went through cognitive restructuring to build a new identity. Most participants described recovery as a non-linear process comprising multiple interactions with health services, relapses, and struggles before eventually identifying as recovered or in recovery.

The triggers for alcohol misuse were diverse and included companionship drinking, occupational stressors, psychological trauma such as childhood abuse or toxic relationships, loss, grief due to the death of a family member or friend, mental health issues, and having surplus income or time. In addition, we identified avoidance behaviour as a strong trigger for alcohol misuse, whereas common turning points were seeking help, having a near-death experience, being informed of having liver disease, changing from negative to positive social connections, self-realisation, and observing others having physical diseases due to alcohol. The co-existing mental health issues were a recurring theme. Anxiety, depression, post-traumatic stress disorder (PTSD), emotional instability, OCD, and anti-social behaviour were commonly reported mental health problems.

### Implications for the ARNCF

The refined framework enhances the understanding of recovery from AUD and poses implications for policy, healthcare practice, and research. Multiple dimensions and types were present across the narratives. The findings demonstrated that recovery from alcohol misuse followed a non-linear path and often required a multiagency approach. This supports the evidence from the original systematic review stating that there are multiple constituent dimensions with associated types and subtypes of alcohol recovery narratives (Subhani et al., 2022). It highlights the need for an integrated healthcare model to effectively mitigate rising alcohol-related harm (Christian & Gilvarry, 1999; Seeling, 2001). It provides more evidence on the importance of regulatory and statutory enforcement policies such as alcohol minimum unit pricing (MUP) at population level to effectively mitigate alcohol-related harm.

The extended ARNCF describes the characteristics of recovery narratives. Future work should explore the application of the framework in larger and more diverse samples to examine any gaps. Recovery narratives have been used to promote and encourage engagement with health services (McGranahan et al., 2019) where they might be used to extend clinical practice. In particular, narratives are a resource for people who are finding recovery challenging (Roe et al., 2020). It can be used to develop material, which supports clinicians in comprehending the subtleties of the recovery journey described by patients during consultations, enhancing the clinician–patient relationship and opening communication channels. In turn, this might enable clinicians to make the most effective choices about treatment, by considering the complex influences sustaining alcohol misuse. The framework can provide structure for future research, policymaking, and the development of personalised interventions for alcohol misuse.

People with drug and alcohol use disorders often suffer from social stigma and stereotyping, and this could be due to inadequate knowledge and awareness and negative attitudes towards patients with AUD (Probst et al., 2015). There is an urgent need for a reference shift away from negative attitudes towards addiction both within the community and among healthcare professionals. In this context, the alcohol recovery narratives can be used to educate the public and healthcare providers to facilitate this reference shift.

### Context and Potential Applications

The notion of narrative psychology can contribute to better understanding of recovery. Sabrin (1986) drew attention to narrative psychology as ‘storied nature of human conduct’, discussing how humans use stories to create meaning and share life experiences. The act of sharing alcohol narratives has been an important component of the AA 12-step programme (Lederman & Menegatos, 2011). In part, the sharing of narratives is important because this method provides the context of personal recovery from addiction, opening opportunity for recognising and working on behaviours in a group setting.

In 2021, there were over 274,000 homeless people in England (Public-Health-England, 2021). This could be a cause or consequence of addictive behaviours (Martijn & Sharpe, 2006). Dame Carol Black’s (2020) independent review concerning illicit drug prevention, treatment, and recovery reported almost a quarter of people engaging with drug and alcohol services have severe housing problems, often associated with higher morbidity (Black, 2020). The current study provides insight into the recovery dynamics of people with a history of homelessness; specifically, the cases suggest increased vulnerability to addictive behaviours and chances of recovery are higher in structured recovery programmes.

The dual diagnosis of AUD and a mental health disorder is a recurring theme and is often related to poor outcomes (Buckley, 2006). The chronic underfunding of drug and alcohol services in the United Kingdom and the lack of provision of integrated healthcare services including mental health services have contributed to a soaring number of alcohol-related harm. A recent audit of drug and alcohol services in Scotland in 2020 showed funding cuts were related to increased drug-related deaths specifically among people from lower socioeconomic backgrounds (McPhee & Sheridan, 2020). Pathological avoidance behaviour can also induce a variety of mental health problems such as anxiety, depression, and PTSD, and the management involves the removal of trigger sources and cognitive restructuring (Treanor & Barry, 2017). Actively addressing mental issues at both policy and practice levels promote longevity of sobriety (Burman, 1997).

### Strengths and Limitations

A pre-existing robust two-stage methodology was adapted (Llewellyn-Beardsley et al., 2020). Participants had diverse socioeconomic characteristics, with a diverse history of homelessness, unemployment, severity of alcohol use disorder, and liver disease stage. The current study suggests developing a conceptual framework can facilitate understanding of narratives and their characteristics in relation to alcohol recovery. Another strength of this study was the collection of data on alcohol consumption and liver stiffness to contextualise narrative information. Future larger studies may wish to examine group differences quantitatively based on these scores.

A limitation of this study is the lack of ethnic diversity which may limit generalisation of results. Our sample was recruited through alcohol treatment services in England, and the majority of people who come into contact with English alcohol and healthcare services identify with a white ethnicity (Subhani et al., 2021). Minority ethnic groups have been identified as disadvantaged in accessing drug and alcohol services (Bayley & Hurcombe, 2010). Future studies should use different methods such as community partners and advocates to include a more diverse sample of people. Further, the current study only recruited participants from the United Kingdom, interviews were conducted in the English language, and none of the included narrators identified as LGBTQIA2S+. The mean age was 56 years which could have an impact on descriptions of drinking trajectory. In this respect, a pool of younger participants may provide novel drinking trajectories and corresponding behaviours.

*‘Religion versus spirituality’* as a type was not identified from these interviews. This may have been due to the under-representation of narratives from participants who experienced recovery within an AA setting. In this sense, spirituality and religion may have been a catalyst to recovery in the AA model (Subhani et al., 2022). Participants did share experiences of belonging. Due to distinct and divergent behaviours across the cases with regard to sense of belonging, there is potential for further exploration to determine any dimensionality in this regard.

## Conclusion

The study applied the preliminary ARNCF to a new sample of recovery narratives and confirmed these narratives are composed of multiple dimensions each with distinct types and subtypes. The findings demonstrate that recovery from alcohol follows a non-linear path and is often achieved through interactions with multiple services. Consequently, the study highlights the need for an integrated healthcare model involving a multiagency approach to effectively mitigate rising alcohol-related harm, holistically. The validated ARNCF can provide an enhanced approach to inform narrative-based research, policy, practice, and intervention development in the field of drug and alcohol addiction.

## Supplemental Material

Supplemental Material - Application and Extension of the Alcohol Recovery Narratives Conceptual Framework Click here for additional data file. Supplemental Material for Application and Extension of the Alcohol Recovery Narratives Conceptual Framework by Mohsan Subhani, Usman Talat, Holly Knight, Joanne R. Morling, Katy A. Jones, Guruprasad P. Aithal, Stephen D. Ryder and Stefan Rennick-Egglestone in Qualitative Health Research

## References

[bibr1-10497323231197384] Anthony W. A. (1993). Recovery from mental illness: The guiding vision of the mental health service system in the 1990s. Psychosocial Rehabilitation Journal, 16(4), 11–23. 10.1037/h0095655

[bibr2-10497323231197384] Barton S. S. (2008). Using narrative inquiry to elicit diabetes self-care experience in an Aboriginal population. Canadian Journal of Nursing Research Archive, 40(3), 16–36. 10.7939/R34F1MM5W 18947090

[bibr3-10497323231197384] Bayley M. Hurcombe R. (2010). Drinking patterns and alcohol service provision for different ethnic groups in the UK: A review of the literature. Ethnicity and Inequalities in Health and Social Care, 3(4), 6–17. 10.5042/eihsc.2011.0073

[bibr4-10497323231197384] Benoot C. Hannes K. Bilsen J. (2016). The use of purposeful sampling in a qualitative evidence synthesis: A worked example on sexual adjustment to a cancer trajectory. BMC Medical Research Methodology, 16(1), 21. 10.1186/s12874-016-0114-6 26891718 PMC4757966

[bibr5-10497323231197384] Best D. Beckwith M. Haslam C. Alexander Haslam S. Jetten J. Mawson E. Lubman D. I. (2016). Overcoming alcohol and other drug addiction as a process of social identity transition: The social identity model of recovery (SIMOR). Addiction Research and Theory, 24(2), 111–123. 10.3109/16066359.2015.1075980

[bibr6-10497323231197384] Best D. W. Lubman D. I. (2012). The recovery paradigm - a model of hope and change for alcohol and drug addiction. Australian Family Physician, 41(8), 593–597. https://www.racgp.org.au/afp/2012/august/the-recovery-paradigm/23145400

[bibr7-10497323231197384] Black D. C. (2020). Independent review of drugs: Prevention, treatment and recovery. The National Archives. Kew, London TW94DU. Retrieved from.https://www.gov.uk/government/publications/review-of-drugs-phase-one-report/review-of-drugs-summary.- contents.

[bibr8-10497323231197384] Blumer H. (1954). What is wrong with social theory? American Sociological Review, 19(1), 3–10. 10.2307/2088165

[bibr9-10497323231197384] Boylstein C. Rittman M. Hinojosa R. (2007). Metaphor shifts in stroke recovery. Health Communication, 21(3), 279–287. 10.1080/10410230701314945 17567259

[bibr10-10497323231197384] Buckley P. F. (2006). Prevalence and consequences of the dual diagnosis of substance abuse and severe mental illness. The Journal of Clinical Psychiatry, 67(Suppl 7), 5–9. 10.4088/jcp.0706e01 16961418

[bibr11-10497323231197384] Burman S. (1997). The challenge of sobriety: Natural recovery without treatment and self-help groups. Journal of Substance Abuse, 9(1997), 41–61. 10.1016/S0899-3289(97)90005-5 9494938

[bibr12-10497323231197384] Butina M. (2015). A narrative approach to qualitative inquiry. American Society for Clinical Laboratory Science, 28(3), 190–196. 10.29074/ascls.28.3.190

[bibr13-10497323231197384] Christian J. Gilvarry E. (1999). Specialist services: The need for multi-agency partnership. Drug and Alcohol Dependence, 55(3), 265–274. 10.1016/S0376-8716(99)00021-6 10428366

[bibr14-10497323231197384] Clandinin D. J. Caine V. (2013). Narrative inquiry. In Reviewing qualitative research in the social sciences (pp. 178–191). Routledge.

[bibr15-10497323231197384] Coyne I. T. (1997). Sampling in qualitative research. Purposeful and theoretical sampling; merging or clear boundaries? Journal of Advanced Nursing, 26(3), 623–630. 10.1046/j.1365-2648.1997.t01-25-00999.x 9378886

[bibr16-10497323231197384] Haas E. (2012). Adolescent perceptions of living with crohn's disease. Dalhousie University Halifax, Nova Scotia.

[bibr17-10497323231197384] Jabareen Y. (2009). Building a conceptual framework: Philosophy, definitions, and procedure. International Journal of Qualitative Methods, 8(4), 49–62. 10.1177/160940690900800406

[bibr18-10497323231197384] Jordens C. F. Little M. Paul K. Sayers E.-J. (2001). Life disruption and generic complexity: A social linguistic analysis of narratives of cancer illness. Social Science and Medicine, 53(9), 1227–1236. 10.1016/s0277-9536(00)00422-6 11556612

[bibr19-10497323231197384] Lederman L. C. Menegatos L. M. (2011). Sustainable recovery: The self-transformative power of storytelling in alcoholics anonymous. Journal of Groups in Addiction and Recovery, 6(3), 206–227. 10.1080/1556035X.2011.597195

[bibr20-10497323231197384] Llewellyn-Beardsley J. Rennick-Egglestone S. Bradstreet S. Davidson L. Franklin D. Hui A. McGranahan R. Morgan K. Pollock K. Ramsay A. Smith R. Thornicroft G. Slade M. (2020). Not the story you want? Assessing the fit of a conceptual framework characterising mental health recovery narratives. Social Psychiatry and Psychiatric Epidemiology, 55(3), 295–308. 10.1007/s00127-019-01791-x 31654089 PMC7612149

[bibr21-10497323231197384] Llewellyn-Beardsley J. Rennick-Egglestone S. Callard F. Crawford P. Farkas M. Hui A. Manley D. McGranahan R. Pollock K. Ramsay A. Sælør K. T. Wright N. Slade M. (2019). Characteristics of mental health recovery narratives: Systematic review and narrative synthesis. PLoS One, 14(3), Article e0214678. 10.1371/journal.pone.0214678 30921432 PMC6438542

[bibr22-10497323231197384] Manning M. L. (2006). Improving clinical communication through structured conversation. Nursing Economic$, 24(5), 268–271. https://pubmed.ncbi.nlm.nih.gov/17131620/17131620

[bibr23-10497323231197384] Martijn C. Sharpe L. (2006). Pathways to youth homelessness. Social Science and Medicine, 62(1), 1–12. 10.1016/j.socscimed.2005.05.007 15985321

[bibr24-10497323231197384] McAdams D. P (1993). The stories we live by: Personal myths and the making of the self. Guilford Press.

[bibr25-10497323231197384] McGranahan R. Rennick-Egglestone S. Ramsay A. Llewellyn-Beardsley J. Bradstreet S. Callard F. Priebe S. Slade M. (2019). Curation of mental health recovery narrative collections: Systematic review and qualitative synthesis. JMIR Mental Health, 6(10), Article e14233. 10.2196/14233 31588912 PMC6915799

[bibr26-10497323231197384] McPhee I. Sheridan B. (2020). AUDIT Scotland 10 years on: Explaining how funding decisions link to increased risk for drug related deaths among the poor. Drugs and Alcohol Today, 20(4), 313–322. 10.1108/DAT-05-2020-0024

[bibr27-10497323231197384] Neale J. Tompkins C. Wheeler C. Finch E. Marsden J. Mitcheson L. Rose D. Wykes T. Strang J. (2015). You’re all going to hate the word ‘recovery’ by the end of this": Service users’ views of measuring addiction recovery. Drugs: Education, Prevention and Policy, 22(1), 26–34. 10.3109/09687637.2014.947564

[bibr28-10497323231197384] Pennebaker J. W. Seagal J. D. (1999). Forming a story: The health benefits of narrative. Journal of Clinical Psychology, 55(10), 1243–1254. 10.1002/(SICI)1097-4679(199910)55:10<1243::AID-JCLP6>3.0.CO;2-N(199910)55:10<1243::Aid-jclp6>3.0.Co;2-n 11045774

[bibr29-10497323231197384] Probst C. Manthey J. Martinez A. Rehm J. (2015). Alcohol use disorder severity and reported reasons not to seek treatment: A cross-sectional study in European primary care practices. Substance Abuse Treatment, Prevention, and Policy, 10(1), 32. 10.1186/s13011-015-0028-z 26264215 PMC4534056

[bibr30-10497323231197384] Public-Health-England . (2021). Rough sleeping in the UK: 2002 to 2021. Office for National Statistics, England, UK. Retrieved from.https://www.ons.gov.uk/peoplepopulationandcommunity/housing/articles/roughsleepingintheuk/2002to2021 (Date accessed 01 01 2023).

[bibr31-10497323231197384] Rennick-Egglestone S. Morgan K. Llewellyn-Beardsley J. Ramsay A. McGranahan R. Gillard S. Hui A. Ng F. Schneider J. Booth S. Pinfold V. Davidson L. Franklin D. Bradstreet S. Arbour S. Slade M. (2019a). Mental health recovery narratives and their impact on recipients: Systematic review and narrative synthesis. Canadian Journal of Psychiatry. Revue Canadienne de Psychiatrie, 64(10), 669–679. 10.1177/0706743719846108 31046432 PMC6783672

[bibr32-10497323231197384] Rennick-Egglestone S. Morgan K. Llewellyn-Beardsley J. Ramsay A. McGranahan R. Gillard S. Hui A. Ng F. Schneider J. Booth S. Pinfold V. Davidson L. Franklin D. Bradstreet S. Arbour S. Slade M. (2019b). Mental health recovery narratives and their impact on recipients: Systematic review and narrative synthesis. Canadian Journal of Psychiatry. Revue Canadienne de Psychiatrie, 64(10), 669–679. 10.1177/0706743719846108 31046432 PMC6783672

[bibr33-10497323231197384] Rennick-Egglestone S. Subhani M. Knight H. Jones K. A. Hutton C. Jackson T. Hutton M. Wragg A. Morling J. R. Sprange K. Ryder S. (2023). Service user and healthcare staff influence in the development of a complex intervention for alcohol misuse: The experience of the KLIFAD study. JMIR Preprints.

[bibr34-10497323231197384] Roe J. Brown S. Yeo C. Rennick-Egglestone S. Repper J. Ng F. Llewelyn-Beardsley J. Hui A. Cuijpers P. Thornicroft G. Manley D. Pollock K. Slade M. Thornicroft G. (2020). Opportunities, enablers, and barriers to the use of recorded recovery narratives in clinical settings. Frontiers in Psychiatry, 11(2020), 1–11. 10.3389/fpsyt.2020.589731 33192738 PMC7661955

[bibr49-10497323231197384] Sarbin T. R. (1986). Narrative psychology: The storied nature of human conduct. Westport, CT, US: Praeger Publishers/Greenwood Publishing Group. xviii, 303-xviii, p3–21.

[bibr35-10497323231197384] Seeling C. King C. Metcalfe E. Tober G. Bates S. (2001). Arrest ReferralÂa proactive multi-agency approach. Drugs: Education, Prevention and Policy, 8(4), 327–333. 10.1080/09687630110048070

[bibr36-10497323231197384] Sharf B. F. (2001). Out of the closet and into the legislature: Breast cancer stories. Health Affairs, 20(1), 213–218. 10.1377/hlthaff.20.1.213 11194844

[bibr37-10497323231197384] Slade M. Amering M. Farkas M. Hamilton B. O'Hagan M. Panther G. Perkins R. Shepherd G. Tse S. Whitley R. (2014). Uses and abuses of recovery: Implementing recovery-oriented practices in mental health systems. World Psychiatry: Official Journal of the World Psychiatric Association (WPA), 13(1), 12–20. 10.1002/wps.20084 24497237 PMC3918008

[bibr38-10497323231197384] Sommer R. (2003). The use of autobiography in psychotherapy. Journal of Clinical Psychology, 59(2), 197–205. 10.1002/jclp.10146 12552628

[bibr39-10497323231197384] Spector-Mersel G. Knaifel E. (2018). Narrative research on mental health recovery: Two sister paradigms. Journal of Mental Health (Abingdon, England), 27(4), 298–306. 10.1080/09638237.2017.1340607 28648112

[bibr40-10497323231197384] Subhani M. Enki D. G. Knight H. Jones K. A. Sprange K. Rennick-Egglestone S. Morling J. R. Wragg A. Hutton C. Ryder S. D. Ryder S. D. (2023). Does knowledge of liver fibrosis affect high-risk drinking behaviour (KLIFAD): An open-label pragmatic feasibility randomised controlled trial. EClinicalMedicine, 61(July 2023), 1–13. 10.1016/j.eclinm.2023.102069 PMC10336239 37448808

[bibr41-10497323231197384] Subhani M. Jones K. A. Sprange K. Rennick-Egglestone S. Knight H. Morling J. R. Wragg A. Ryder S. D. Enki D. G. (2021). Does knowledge of liver fibrosis affect high-risk drinking behaviour (KLIFAD)? Protocol for a feasibility randomised controlled trial. BMJ Open, 11(11), Article e054954. 10.1136/bmjopen-2021-054954 PMC8572412 34732502

[bibr42-10497323231197384] Subhani M. Sheth A. Unitt S. Aithal G. P. Ryder S. D. Morling J. R. (2021). The effect of covid-19 on alcohol use disorder and the role of universal alcohol screening in an inpatient setting: A retrospective cohort control study. Alcohol and Alcoholism, 57(2), 203–210. 10.1093/alcalc/agab059 PMC8499734 34423352

[bibr43-10497323231197384] Subhani M. Talat U. Knight H. Morling J. R. Jones K. A. Aithal G. P. Ryder S. D. Llewellyn-Beardsley J. Rennick-Egglestone S. (2022). Characteristics of alcohol recovery narratives: Systematic review and narrative synthesis. PLoS One, 17(5), Article e0268034. 10.1371/journal.pone.0268034 35511789 PMC9070949

[bibr44-10497323231197384] Treanor M. Barry T. J. (2017). Treatment of avoidance behavior as an adjunct to exposure therapy: Insights from modern learning theory. Behaviour Research and Therapy, 96(September 2017), 30–36. 10.1016/j.brat.2017.04.009 28477845

[bibr45-10497323231197384] Weegmann M. Piwowoz-Hjort E. (2009). ‘Naught but a story’: Narratives of successful AA recovery. Health Sociology Review, 18(3), 273–283. 10.5172/hesr.2009.18.3.273

[bibr46-10497323231197384] Witkiewitz K. Wilson A. D. Pearson M. R. Montes K. S. Kirouac M. Roos C. R. Hallgren K. A. Maisto S. A. Maisto S. A. (2019). Profiles of recovery from alcohol use disorder at three years following treatment: Can the definition of recovery be extended to include high functioning heavy drinkers? Addiction, 114(1), 69–80. 10.1111/add.14403 30063267 PMC6289769

[bibr47-10497323231197384] Yaskowich K. M. Stam H. J. (2003). Cancer narratives and the cancer support group. Journal of Health Psychology, 8(6), 720–737. 10.1177/13591053030086006 14670206

[bibr48-10497323231197384] Zwerenz R. Becker J. Knickenberg R. J. Siepmann M. Hagen K. Beutel M. E. (2017). Online self-help as an add-on to inpatient psychotherapy: Efficacy of a new blended treatment approach. Psychotherapy and Psychosomatics, 86(6), 341–350. 10.1159/000481177 29131090

