# Supplementary material for: Application and Extension of the Alcohol Recovery Narratives Conceptual Framework
Source: Qual Health Res. 2023 Sep 8;33(13):1203–17. doi: 10.1177/10497323231197384 (PMC10626984; doi:10.1177/10497323231197384)
Supplement: Supplemental Material - Application and Extension of the Alcohol Recovery Narratives Conceptual Framework [file sj-pdf-1-qhr-10.1177_10497323231197384.pdf]

S1 Text. Recovery Definitions

A recovery in mental health has been define as;

"A deeply personal, unique process of change, a way of living a satisfying, hopeful and contributing life even with limitations caused by illness [and] a process involving the development of new meaning or purpose in one's life" (Anthony, 1993; Rennick-Egglestone et al., 2019).

In context of Alcohol use disorder (AUD) recovery has been defined in variety of ways; Katie Wiltkiewitz et al recognise recovery in AUD as a period of sustained abstinence from alcohol and improvement in social and employment functioning, and mental and physical health (Witkiewitz et al., 2019).

The UK Drug Policy commission define recovery in substance misuse as ''voluntarily sustained control over substance use, which maximises health and wellbeing and participation in the rights, roles and responsibilities of society'.

The Betty ford institute consensus panel define recovery in Alcohol and substance misuse as 'a voluntarily maintained lifestyle composed characterized by sobriety, personal health, and citizenship' . The components of this definition are elaborated as; **Sobriety:** refers to abstinence from alcohol and all other nonprescribed drugs. **Personal health:** refers to improved quality of personal life as defined and measured by validated instruments such as the physical health, psychological health, independence, and spirituality scales of the World Health Organization QOL instrument. **Citizenship:** Citizenship refers to living with regard and respect for those around you as defined and measured by validated instruments such as the social function and environment scales of the WHO-QOL instrument (Betty-Ford-Institute-Consensus-Panel, 2007).

Whereas U.S. Department of Health & human services Substance abuse and mental health service administration (SAMHSA) define recovery in substance use disorder as 'A process of change through which individuals improve their health and wellness, live a self-directed life, and strive to reach their full potential'.

S2 Text. explanation of subtypes for 'Alcohol environment'

'Affordability' describes the buying capacity of a person to afford alcohol. One can consume more alcohol if purchasing power is increased or otherwise can turn to cheap alternatives if purchasing power decreases. 'Acceptability' describes the social acceptance of different drinking behaviours, specifically, our participants found that certain factors within a culture supported detrimental levels of alcohol consumption to the extent that was experienced as a normal behaviour validated by others. 'Availability' describes the ease of access to alcohol. Our participants narrated that over the years alcohol has become easier to access due to policies such as changes in supermarket alcohol licensing and seasonal deals, allowing alcohol sales outside pubs with extended hours of business, and the adjacent propensity of drinking at home. 'Exposure and advertisement' describe how alcohol has been promoted through multimedia, at times ambiguously labelled, or often lacking details concerning the harmful impact of alcohol on health. 'Constrained services' describes the pressure on health services to deal with alcohol misuse and associated mental health problems in a timely manner, which can result in reduced trust among service users. The 'Pandemic' describes the impact of the Coronavirus outbreak and imposed social restrictions on drinking behaviours. Participants described their alcohol consumption as increased during the pandemic. 'Early detection of liver disease' describes the impact of having non-invasive tests (NITs) of liver disease and knowing the results. Participants narrated that by knowing the results of transient elastography supported them to change addictive behaviour. The illustrative examples for 'Alcohol environment' as a dimension are given in the S3 Table.

S1 Table. Sample interview guide

<u>What was life for you before you started using alcohol?</u>

What triggered you to drink harmfully?
What was your social setup like?
How much you were drinking at peak ?

<u>What does recovery mean to you?</u>

What gives you hope?
What makes you feel well?

<u>What has helped your recovery?</u>

What was your first step on your recovery journey?
What helped you take this step?
What works for you and why?
What activities have helped you?
How do you feel when you are doing them?
What has helped during times of hardship?
At what point did you realise that you needed support?
Where did you find the support? Was this challenging?
Who has supported you during your recovery journey?
What were the barriers to recovery?
How did you overcome them?
What has been unhelpful or missing in your recovery?

<u>What have you learned about recovery?</u>

Do you have any techniques that have been helpful when you are feeling down?

What sort of lessons would you like to pass onto others?

If you could give one thing to assist someone's recovery what would that be?

What has helped you to build resilience?
What would you tell someone who feels they won't recover?
How did you deal with changes to your recovery journey?

This was sample interview guide used for semi-structured interviews

S2 Table. Illustrative examples for alcohol and environment

	Quotes from narratives
Affordability	I started work and one of the things that changed about drinking was that up to the 70s basically, if you wanted to drink you went to a pub, if you wanted to buy a bottle of beer to drink at home, you went to the pub and you bought it and it cost a lot of money. So, drinking at home wasn't really very much of an option. While money was a bit tighter but also you could buy cheap alcohol in the supermarket
Acceptability	It's a big part of our culture. English, British culture. I've had numerous discussions with my partner about this and erm, Britain as a country and as a society has, I think a very unhealthy attitude to alcohol.
Availability	The other problem is it's so readily available. It got to the point where you go into the supermarkets, they're on offer, so I would get a box.
Exposure and advertisement	I think people don't take a blind bit of notice and I think also on the packaging I mean the unit thing is, you look on a packet of cigarettes and it's the whole packet. But there's no advert with the poor, sad alcohol-dependent grandad, brother, uncle, sister, auntie
Constrained services	And I've been in touch and on the books with the mental health system for years, and they agreed that I have issues and need some help with them. Erm, but they wouldn't touch me because I was drinking, they said it was a dangerous road to go down,
Pandemic	During the lockdown I wasn't, but I used to go to car boots and things, so I'd have somewhere to go, say a Wednesday, Saturday, Sunday, but I won't go near the motor if I've been drinking
Early detection of liver disease	It wasn't until the FibroScan that I realised how much damage I had actually done and how little time I had left, so check it out kids

The quotes are taken from participants' interviews.

References

Anthony, WA (1993) Recovery from mental illness: The guiding vision of the mental health service system in the 1990s. *Psychosocial Rehabilitation Journal* **16**: 11-23.

Betty-Ford-Institute-Consensus-Panel (2007) What is recovery? A working definition from the Betty Ford Institute. *Journal of substance abuse treatment* **33**: 221-8.

Rennick-Egglestone, S, Morgan, K, Llewellyn-Beardsley, J *et al.* (2019) Mental Health Recovery Narratives and Their Impact on Recipients: Systematic Review and Narrative Synthesis. *Canadian journal of psychiatry Revue canadienne de psychiatrie* **64**: 669-79.

Witkiewitz, K, Wilson, AD, Pearson, MR *et al.* (2019) Profiles of recovery from alcohol use disorder at three years following treatment: can the definition of recovery be extended to include high functioning heavy drinkers? *Addiction* **114**: 69-80.
